# Circulating long noncoding RNAs H19 and GAS5 are associated with type 2 diabetes but not with diabetic retinopathy: A preliminary study

**DOI:** 10.17305/bjbms.2019.4533

**Published:** 2020-08

**Authors:** Manal S. Fawzy, Ahmed A. Abdelghany, Eman A. Toraih, Abeer M. Mohamed

**Affiliations:** 1 Department of Medical Biochemistry and Molecular Biology, Faculty of Medicine, Suez Can al University, Ismailia, Egypt; 2 Department of Biochemistry, Faculty of Medicine, Northern Border University, Arar, Saudi Arabia; 3 Department of Ophthalmology, Faculty of Medicine, Suez Canal University, Ismailia, Egypt; 4 Department of Surgery, School of Medicine, Tulane University, New Orleans, Louisiana, USA; 5 Department of Histology and Cell Biology (Genetics Unit), Faculty of Medicine, Suez Canal University, Ismailia, Egypt; 6 Department of Clinical Pathology and Clinical Chemistry, Faculty of Medicine, Sohag University, Sohag, Egypt; 7 Department of Clinical Laboratory Sciences, Al-Ghad International College for Applied Medical Sciences, Abha, Saudi Arabia

**Keywords:** Long noncoding RNA, H19, GAS5, diabetic retinopathy, aflibercept, type 2 diabetes mellitus, T2DM

## Abstract

Recently, a wide range of biological and pathological roles of long noncoding RNAs (lncRNAs) have been discovered. However, the potential role of circulating lncRNAs H19 and GAS5 in type 2 diabetes mellitus (T2DM) and diabetic retinopathy (DR) is not clear. Here, we assessed the plasma levels of H19 and GAS5 lncRNAs in T2DM patients with/without DR and evaluated if H19 and GAS5 pre-treatment plasma levels are a predictor of early response to a single aflibercept dose in DR subgroup. Plasma lncRNA expression profiles of 119 T2DM patients (66 with DR and 53 without DR) and 110 healthy controls were determined by quantitative reverse transcription PCR. The association of lncRNA expression profiles with clinical features and aflibercept early response in DR patients was investigated. Relative H19 expression levels were significantly increased in T2DM group (including DR and non-DR subgroups) vs. controls, while GAS5 levels were decreased in T2DM group (*p* < 0.001). There was no significant difference in H19 and GAS5 expression levels between DR and non-DR subgroups. H19 and GAS5 expression profiles were not significantly correlated with clinical parameters or response to aflibercept therapy in DR subgroup. Our findings indicate that the circulating lncRNAs H19 and GAS5 may be associated with T2DM prevalence but may not have an important diagnostic/prognostic role in DR or early response to aflibercept intravitreal injection in DR patients. Large-scale transcriptomic studies are warranted to validate our results and investigate other lncRNA candidates in T2DM.

## INTRODUCTION

As a common microvascular complication of diabetes, diabetic retinopathy (DR) is a major cause of vision loss, with considerable social and economic impact worldwide [1]. The prevalence of DR is continuing to increase as a result of the longer duration of diabetes due to the increased lifespan of diabetic patients [2]. It has been reported that 28.8% of type 2 diabetes mellitus (T2DM) patients develop DR, whereas 22.2% do not develop DR regardless of glycemic exposure, indicating that genetic factors may influence DR development [3].

Genetic and epigenetic mechanisms underlying DR are increasingly being explored [4]. Several studies have shown that long noncoding RNAs (lncRNAs) may have a role in the development of T2DM and associated outcomes [5,6]. In particular, lncRNAs were implicated in critical cellular processes such as alterations of extracellular matrix proteins, angiogenesis and inflammatory insult, which can contribute to fibrosis and retinal tissue damage with subsequent ocular complications [7,8]. Hence, dysregulated expression of lncRNAs may be used a biomarker in clinical diagnosis, prognosis, and therapy of many metabolic disorders and complications, including DR [9].

Current data support that the imprinted maternally expressed H19 gene that encodes a lncRNA affects glucose metabolism in muscle cells [10], gluconeogenesis, and hepatic glucose output [11]. Furthermore, recent *in vitro* and *in vivo* studies confirmed a role of H19 lncRNA in targeting several molecular pathways that could be related to DR pathogenesis. For example, Thomas et al. [12] indicated H19 to participate in the regulation of endothelial-mesenchymal transition (EMT) in the diabetic retina through several mechanisms, including the positive regulation of microRNA-200b, which is one of the highly characterized angiomiRs involved in angiogenesis [13]. H19 is also suggested to participate in hypoxia-induced stress response via regulating NADPH oxidase 4 (NOX4) and endothelial nitric oxide synthase/nitric oxide (eNOS/NO) signaling [14].

The serum level of another lncRNA, growth arrest-specific transcript 5 (GAS5), has been correlated with T2DM prevalence [15] and its downregulation has been associated with insulin resistance [16]. Recently, GAS5 has also been implicated in maintaining retinal ganglion cell survival in glaucoma via the activation of transforming growth factor beta (TGF-β) pathway, which induces cell proliferation and differentiation [17].

In the current study, we explored the potential role of H19 and GAS5 lncRNAs in T2DM and DR considering the following: 1) their implication in DM- and DR-related cellular pathways; 2) to the best of our knowledge, the circulating levels of H19 and GAS5 have not been evaluated in DM/DR in our population before; 3) although the levels of circulating lncRNAs have been associated with the development of DR [18,19], most of the previous studies focused on diabetic rat models or endothelial cells cultured in high-glucose conditions [12,20,21]. Here, we compared the plasma levels of circulating H19 and GAS5 lncRNAs of T2DM patients (with/without DR) and healthy controls. In addition, as circulating lncRNAs could be used as novel non-invasive biomarkers for early prediction of DR or response to anti-vascular endothelial growth factor (VEGF) treatment [15], we correlated the pre-treatment levels of the circulating lncRNAs to patient clinical data in DR subgroup, including response to a single dose of anti-VEGF aflibercept intravitreal (IV) injection.

## MATERIALS AND METHODS

### Ethical statement

The study was approved by the Institutional Research Ethics Committee of the Faculty of Medicine, Suez Canal University, No. 3651, and the research was conducted in accordance with the Helsinki Declaration. All participants provided informed consent before taking part in the study.

### Participants

This case-control study enrolled T2DM patients attending ophthalmology outpatient clinics of the Suez Canal University hospitals and Eljawhara Center for Ophthalmic Surgeries and LASIK during the period between November 2018 and the end of February 2019. One hundred and nineteen consecutive and randomly assigned T2DM adult patients with (n = 66) and without (n = 53) confirmed DR diagnosis, according to the Early Treatment Diabetic Retinopathy Study (ETDRS) report [22], and 110 healthy controls were recruited. The characteristics of the patient and control group with their exclusion criteria are detailed in our previous work [23].

### Clinical evaluation

All participants were subjected to a thorough ophthalmic evaluation, which included the best-corrected visual acuity (BCVA) using the Logarithm of the Minimum Angle of Resolution (LogMAR) at the initial presentation and, in the subsequent follow-up visits, an optical coherence tomography (OCT). Aflibercept IV injection was scheduled for DR subgroup. Based on the results of the initial examination by fundus fluorescein angiography (FFA), the type and classification (focal vs. diffuse) of maculopathy were determined by an ophthalmologist. The ischemic type was excluded to avoid unreliable results. DR classification into non-proliferative DR (NPDR) and proliferative DR (PDR) was done according to the ETDRS [22,24]. Subsequently, NPDR was classified into mild, moderate, and severe. Macular edema diagnosis was based on both clinical and OCT findings, as described previously [22].

The patients’ retinas were subjected to evaluation by OCT using OPTOVUE RTvue XR (OPTOVUE Inc., Fremont, CA, USA) to measure the central macular thickness (CMT). Additionally, a map of fast macular thickness, centered at the fovea, was done [23].

OCT findings of diabetic macular edema (DME) included Central Subfield Thickness (CST) more than 290 µm together with spongy intraretinal edema. Cases with an interrupted inner segment/outer segment (IS/OS) junction or interrupted external limiting membrane (ELM) were excluded, as they have a poor prognosis that would yield invalid results. All patients with DME were subjected to IV injection (2 mg in 0.05 mL) of aflibercept (Eylea 40 mg/mL, Bayer Pharma AG, Berlin, Germany) [25], preceded by blood sampling. Patient follow-up was scheduled for the 1^st^ day, one week, and one month post-IV injection. Second check using the BCVA and OCT was performed after four weeks to assess the CMT. In line with our aim to evaluate the association of the selected lncRNAs with the initial response to a single dose of aflibercept, the improvement of BCVA more than 2 lines of the Snellen chart (converted to LogMAR units for statistical analysis) and the reduction of CST more than 15% of the pre-treatment thickness were considered as a response to treatment. Continuous follow-up of patients with recorded BCVA and CMT changes according to the standard schedules and reinjections were applied.

### Quantitative reverse transcription polymerase chain reaction (qRT-PCR) for H19 and GAS5

Total RNA was isolated from (400 µl) plasma by QIAzol Lysis Reagent (Qiagen, Hilden, Germany) according to the modified protocol of Qiagen RNeasy Kit (Cat. No.74104, Qiagen, Hilden, Germany). Extracted RNA was subjected to integrity check and concentration determination by gel electrophoresis and NanoDrop ND-1000 spectrophotometer (NanoDrop Tech., Inc. Wilmington, DE, USA) at the absorbance ratio 260/280 nm, respectively. Extracted RNA was subsequently subjected to reverse transcription using High Capacity cDNA Reverse Transcription Kit (Applied Biosystems, USA; P/N 4368814), as described previously [23]. Each run included glyceraldehyde 3-phosphate dehydrogenase (GAPDH) quantification for data normalization and appropriate negative controls (i.e., no template control [NTC] and no reverse transcriptase control [NRT]). Specific TaqMan assay for each specified lncRNA that consists of a pair of PCR primers, a sequence-specific TaqMan probe with a dye label (FAM) on the 5′ end, a minor groove binder (MGB), and non-fluorescent quencher (NFQ) on the 3′ end (Thermo Fisher Scientific, Applied Biosystems, TaqMan Noncoding RNA assays) was run in duplicate on StepOne™ Real-Time PCR System (Applied Biosystems, Foster City, California, USA) following a protocol described in detail previously [26]. All PCR reactions were carried out in accordance with the Minimum Information for Publication of Quantitative Real-Time PCR Experiments (MIQE) guidelines [27].

### Statistical analysis

Statistical analysis was performed using IBM SPSS Statistics for Windows, Version 23.0. (IBM Corp., Armonk, NY) and GraphPad Prism version 7.00 for Windows (GraphPad Software, La Jolla California USA). Chi-square, Fisher’s exact, student’s t, and Mann-Whitney U tests were employed for comparisons between study groups according to data distribution. Bivariate correlation analysis was applied using the Spearman’s correlation. The fold change of the expression of lncRNAs in each patient relative to the controls was quantified based on the quantification cycle (Cq) value and the 2^−ΔΔCq^ method [28]. Values of *p* < 0.05 were considered statistically significant.

## RESULTS

### Clinical characteristics of study participants

The study included 119 patients (87 females and 32 males; mean age of 61 ± 8.84) and 110 controls (83 females and 27 males; mean age of 60.5 ± 10.7), with comparable mean age (*p* = 0.748) and sex ratio (*p* = 0.763). Among diabetic patients, 66 had DR (45 women and 21 men), while 53 of the patients (42 women and 11 men) did not have ophthalmological problems. No history of other concomitant diabetic complications was observed. As expected, DR patients had a more prolonged duration (years) of diabetes (15.8 ± 7.33) compared to non-DR patients [12.2 ± 4.4] (*p* = 0.002). In addition, insulin dependency was more common among DR patients (*p* = 0.003, OR = 3.58, 95% CI = 1.54 to 8.31).

**FIGURE 1:**
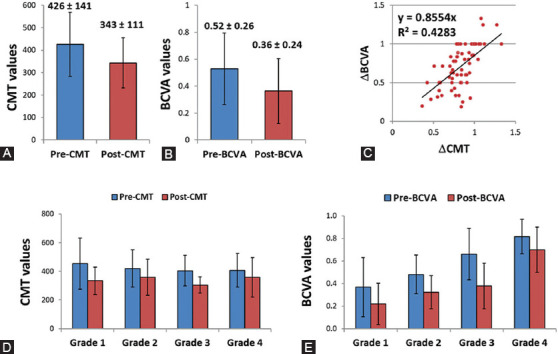
Ophthalmological response in DR subgroup after intraocular anti-VEGF injection. (A) The mean CMT values declined in post-treatment group compared with pre-treatment group. (B) The mean BCVA values declined in post-treatment group compared with pre-treatment group. (C) Correlation analysis of change of CMT and BCVA. (D) There was no significant difference between patients regarding the CMT values classified by DR grade; Grade 1: Mild NPDR, Grade 2: Moderate NPDR, Grade 3: Severe NPDR, Grade 4: PDR. (E) There was no significant difference between patients regarding the BCVA values classified by DR grade (1, 2, 3, and 4). VEGF: Vascular endothelial growth factor; CMT: Central macular thickness; BCVA: Best-corrected visual acuity; DR: Diabetic retinopathy; NPDR: Non-proliferative diabetic retinopathy; PDR: Proliferative diabetic retinopathy; Pre: Prior treatment with aflibercept; Post: After treatment with aflibercept.

The mean baseline CMT and BCVA values of DR patients were 426 ± 141 and 0.52 ± 0.2, respectively. The mean post-treatment values declined to 343 ± 111 and 0.36 ± 0.2, respectively. There was no significant difference between patients with different grades (Figure 1). When DR subgroup was classified by ∆CMT, 4/5 of patients exhibited a remarkable improvement in the CMT one month after intraocular anti-VEGF drug injection. This drug responder subgroup had a significantly different CMT (*p* < 0.001) and BCVA (*p* < 0.001) values compared to non-responders (Table 1). CMT change was moderately correlated with the degree of change of BCVA [r = 0.598, *p* < 0.001] (Figure 1C).

**TABLE 1:**
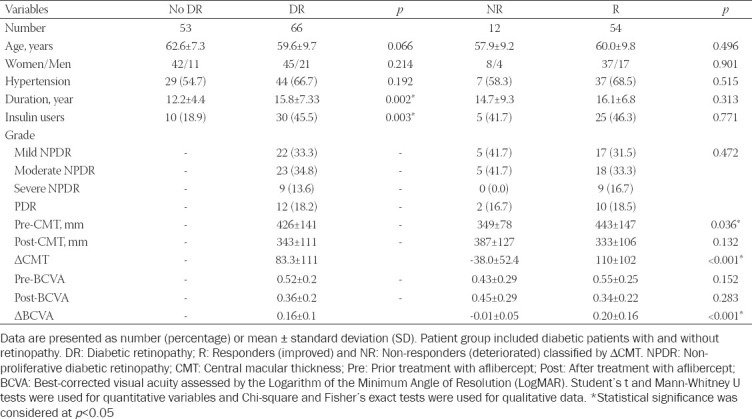
Clinical characteristics of patients with type 2 diabetes mellitus (T2DM)

### LncRNA expression

**FIGURE 2:**
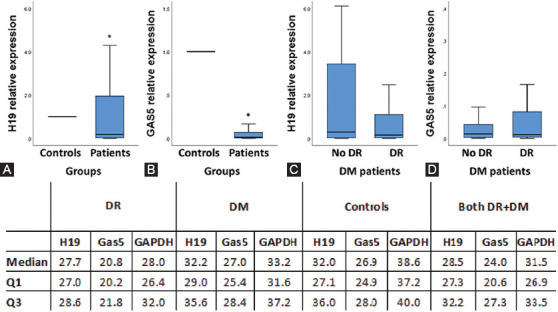
Expression levels of H19 and GAS5 lncRNAs in the study population. (A) The relative expression of H19 was significantly upregulated in diabetic patients compared to controls. (B) The relative expression of GAS5 was significantly downregulated in diabetic patients compared to controls. (C) H19 plasma relative expression levels were comparable between patients with and without DR. (D) GAS5 plasma relative expression levels were comparable between patients with and without DR. The expression level of each lncRNA was detected by quantitative reverse transcription PCR. The data output is expressed as a fold-change and normalized to GAPDH levels. Mann-Whitney U test was used. The control level is set at relative expression of 1.0. LncRNA: Long noncoding RNA; GAPDH: Glyceraldehyde 3-phosphate dehydrogenase; DR: Diabetic retinopathy; No DR: Diabetic patients without DR; DM: Diabetes mellitus; Q1/Q3: The first/third quartile. *Compared to controls; the statistical significance was considered at *p* < 0.05.

The relative expression of H19 was significantly upregulated in diabetic patients compared to controls (*p* < 0.001), while GAS5 was downregulated in the patient group [*p* < 0.001] (Figure 2A and B). The expression profiles did not show a significant association with clinical parameters, including glycated hemoglobin (HbA1c) blood levels, which reflects the state of diabetes control and the early response to single-dose anti-VEGF treatment (Table 2). In addition, H19 and GAS5 plasma levels were comparable between patients with and without DR (Figure 2C and D).

**TABLE 2:**
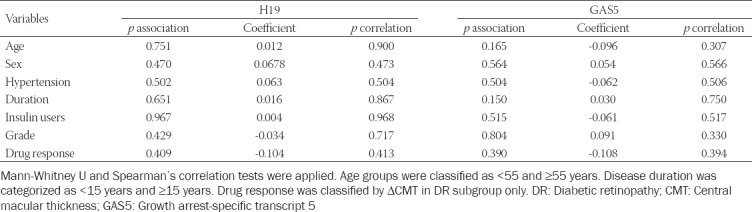
Association between lncRNA expression and clinical features of T2DM patients

## DISCUSSION

Poor outcomes of diabetes remain one of the critical challenges that impose a substantial socioeconomic burden on countries. As previously stated “Understanding the bio-molecular events underlying diabetes could provide new effective diagnostic and therapeutic tools to combat the disease.” [29].

It is important to recognize the role of lncRNAs in DM/DR development and progression. The present study is the first to demonstrate dysregulation of H19 and GAS5 circulating RNAs in diabetic patients with and without retinopathy compared to controls in a population from the Middle East region. The pre-treatment level of H19 and GAS5 circulating lncRNA was upregulated and downregulated, respectively in T2DM patients compared to controls. This finding is consistent with recent reports showing a correlation of lncRNA dysregulation with diabetes and its complications [5,6,15] and suggesting the implication of lncRNAs in the cellular pathways underlying the disease [9,15].

The lncRNA H19 was found to be involved in the regulation of metabolic changes related to T2DM [30]. Interestingly, the role of lncRNAs to control islet development and function was first demonstrated in a study showing that altered H19 expression is implicated in transgenerational transmission of gestational diabetes and diabetes-related deterioration of islet structure and function during pregnancy in mice [31]. Gao et al. [10] reported decreased H19 expression in skeletal muscle in DM and suggested its role in the impairment of insulin signaling and reduction of glucose uptake. In the Goyal study [11], the inhibition of H19 expression by small interfering RNA (siRNA) in HepG2 cells and primary mouse hepatocytes increased the levels of gluconeogenic gene expression, which was accompanied by an increase in hepatic glucose output. In HepG2 cells, H19 inhibition led to dysfunction of insulin signaling, mediated by increased nuclear localization of the transcriptional regulator forkhead box O1 (FoxO1) [11]. These findings support H19 role in the regulation of physiological hepatic response during diabetes.

DR-related mechanisms such as hyperglycemia-induced oxidative stress and hypoxia may lead to lncRNA upregulation, as lncRNA and protein-coding genes are regulated by similar events [29]. In a recent study involving retinal endothelial cells exposed to glucose and a mouse model of DR, Thomas et al. showed that H19 is involved in EMT in the retina. They also demonstrated hyperglycemia-induced downregulation of H19 in the vitreous humor from individuals with PDR compared to non-diabetic controls [12]. Nevertheless, it is important to note that lncRNAs have cell and tissue-specific expression [32], where they regulate homeostasis and the expression of protein-coding or other noncoding RNA genes [33]. In addition, intracellular levels of lncRNAs are different from those in circulation [15]. In this study, we could not compare the plasma H19 levels of DR patients to the findings of previous studies because they quantified lncRNAs locally either in retinal tissues or in the vitreous humor. In this sense, more studies are warranted to investigate the correlation of circulating lncRNA levels with lncRNA levels in the retina or the vitreous humor.

The downregulation of GAS5 observed in the present study is consistent with the results of Carter et al. [15] who were the first to reveal that a decrease in GAS5 serum levels is correlated with T2DM, in a cohort of US military veterans. Their receiver operating characteristics curve analysis and quantitative PCR results showed that participants with absolute GAS5 < 10 ng/µL have almost twelve times higher odds of having diabetes [15].

The GAS5 gene is located at 1q25, a locus that displays dysregulation in several cancers [34,35]. It has been found that GAS5 suppresses rapamycin, an mTOR (mammalian target of rapamycin) inhibitor, and mTOR is implicated in the regulation of beta-cell mass and consequently in T2DM development [36].

Collectively, the current findings indicate cell-specific and multiple roles of lncRNAs, including H19 and GAS5 lncRNAs. Given the essential regulatory roles of lncRNAs in gene expression, it will be interesting to identify the molecular targets of H19 and GAS5 that are implicated in T2DM [15].

## CONCLUSION

Although we showed that plasma levels of H19 and GAS5 differ significantly between T2DM patients (with and without DR) and healthy controls, the lncRNA levels were not correlated with DR grades or early response to single-dose aflibercept. Therefore, H19 and GAS5 may not have an obvious role in DR or in the prediction of anti-VEGF early response in our population. Further follow-up studies with pre- and post-treatment analysis of circulating lncRNA levels may reveal a higher predictive value of these RNAs for early anti-VEGF treatment response, compared to the cross-sectional analysis. Additionally, future studies using global transcriptome analysis to avoid the subjective bias in the selection of lncRNAs may identify novel lncRNAs with potential prognostic or predictive role in DR.

## References

[1] Ting DS, Cheung GC, Wong TY (2016). Diabetic retinopathy: Global prevalence, major risk factors, screening practices and public health challenges: A review. Clin Exp Ophthalmol.

[2] Deshpande AD, Harris-Hayes M, Schootman M (2008). Epidemiology of diabetes and diabetes-related complications. Phys Ther.

[3] Ma J, Wang J, Liu Y, Wang C, Duan D, Lu N (2017). Comparisons of serum miRNA expression profiles in patients with diabetic retinopathy and type 2 diabetes mellitus. Clinics (Sao Paulo).

[4] Reddy MA, Zhang E, Natarajan R (2015). Epigenetic mechanisms in diabetic complications and metabolic memory. Diabetologia.

[5] Jae N, Dimmeler S (2015). Long noncoding RNAs in diabetic retinopathy. Circ Res.

[6] Goyal N, Kesharwani D, Datta M (2018). Lnc-ing non-coding RNAs with metabolism and diabetes: Roles of lncRNAs. Cell Mol Life Sci.

[7] Biswas S, Sarabusky M, Chakrabarti S (2019). Diabetic retinopathy, lncRNAs, and inflammation: A dynamic, interconnected network. J Clin Med.

[8] Biswas S, Chakrabarti S (2019). Increased extracellular matrix protein production in chronic diabetic complications: Implications of non-coding RNAs. Noncoding RNA.

[9] Leti F, DiStefano JK (2017). Long noncoding RNAs as diagnostic and therapeutic targets in type 2 diabetes and related complications. Genes (Basel).

[10] Gao Y, Wu F, Zhou J, Yan L, Jurczak MJ, Lee HY (2014). The H19/let-7 double-negative feedback loop contributes to glucose metabolism in muscle cells. Nucleic Acids Res.

[11] Goyal N, Sivadas A, Shamsudheen KV, Jayarajan R, Verma A, Sivasubbu S (2017). RNA sequencing of db/db mice liver identifies lncRNA H19 as a key regulator of gluconeogenesis and hepatic glucose output. Sci Rep.

[12] Thomas AA, Biswas S, Feng B, Chen S, Gonder J, Chakrabarti S (2019). lncRNA H19 prevents endothelial-mesenchymal transition in diabetic retinopathy. Diabetologia.

[13] Sinha M, Ghatak S, Roy S, Sen CK (2015). microRNA-200b as a switch for inducible adult angiogenesis. Antioxid Redox Signal.

[14] Zhu Y, Ni T, Lin J, Zhang C, Zheng L, Luo M (2019). Long non-coding RNA H19, a negative regulator of microRNA-148b-3p, participates in hypoxia stress in human hepatic sinusoidal endothelial cells via NOX4 and eNOS/NO signaling. Biochimie.

[15] Carter G, Miladinovic B, Patel AA, Deland L, Mastorides S, Patel NA (2015). Circulating long noncoding RNA GAS5 levels are correlated to prevalence of type 2 diabetes mellitus. BBA Clin.

[16] Lin H, Xing W, Li Y, Xie Y, Tang X, Zhang Q (2018). Downregulation of serum long noncoding RNA GAS5 may contribute to insulin resistance in PCOS patients. Gynecol Endocrinol.

[17] Xu Y, Xing YQ (2018). Long non-coding RNA GAS5 contributed to the development of glaucoma via regulating the TGF-beta signaling pathway. Eur Rev Med Pharmacol Sci.

[18] Sathishkumar C, Prabu P, Mohan V, Balasubramanyam M (2018). Linking a role of lncRNAs (long non-coding RNAs) with insulin resistance, accelerated senescence, and inflammation in patients with type 2 diabetes. Hum Genomics.

[19] Li Q, Pang L, Yang W, Liu X, Su G, Dong Y (2018). Long non-coding RNA of myocardial infarction associated transcript (LncRNA-MIAT) promotes diabetic retinopathy by upregulating transforming growth factor-beta1 (TGF-beta1) signaling. Med Sci Monit.

[20] Yan B, Tao ZF, Li XM, Zhang H, Yao J, Jiang Q (2014). Aberrant expression of long noncoding RNAs in early diabetic retinopathy. Invest Ophthalmol Vis Sci.

[21] Li CP, Wang SH, Wang WQ, Song SG, Liu XM (2017). Long noncoding RNA-Sox2OT knockdown alleviates diabetes mellitus-induced retinal ganglion cell (RGC) injury. Cell Mol Neurobiol.

[22] Early Treatment Diabetic Retinopathy Study Research Group (1991). Early treatment diabetic retinopathy study design and baseline patient characteristics. ETDRS report number 7. Ophthalmology.

[23] Toraih EA, Abdelghany AA, Abd El Fadeal NM, Al Ageeli E, Fawzy MS (2019). Deciphering the role of circulating lncRNAs: RNCR2, NEAT2, CDKN2B-AS1, and PVT1 and the possible prediction of anti-VEGF treatment outcomes in diabetic retinopathy patients. Graefes Arch Clin Exp Ophthalmol.

[24] Zhang X, Chee WK, Liu S, Tavintharan S, Sum CF, Lim SC (2018). Association of plasma osteopontin with diabetic retinopathy in Asians with type 2 diabetes. Mol Vis.

[25] Virgili G, Parravano M, Menchini F, Evans JR (2014). Anti-vascular endothelial growth factor for diabetic macular oedema. Cochrane Database Syst Rev.

[26] Fakhr-Eldeen A, Toraih EA, Fawzy MS (2019). Long non-coding RNAs MALAT1, MIAT and ANRIL gene expression profiles in beta-thalassemia patients: A cross-sectional analysis. Hematology.

[27] Bustin SA, Benes V, Garson JA, Hellemans J, Huggett J, Kubista M (2009). The MIQE guidelines: Minimum information for publication of quantitative real-time PCR experiments. Clin Chem.

[28] Livak KJ, Schmittgen TD (2001). Analysis of relative gene expression data using real-time quantitative PCR and the 2(-Delta Delta C(T)) method. Methods.

[29] Coucha M, Elshaer SL, Eldahshan WS, Mysona BA, El-Remessy AB (2015). Molecular mechanisms of diabetic retinopathy: Potential therapeutic targets. Middle East Afr J Ophthalmol.

[30] Kornfeld JW, Bruning JC (2014). Regulation of metabolism by long, non-coding RNAs. Front Genet.

[31] Ding GL, Wang FF, Shu J, Tian S, Jiang Y, Zhang D (2012). Transgenerational glucose intolerance with Igf2/H19 epigenetic alterations in mouse islet induced by intrauterine hyperglycemia. Diabetes.

[32] Wu Z, Liu X, Liu L, Deng H, Zhang J, Xu Q (2014). Regulation of lncRNA expression. Cell Mol Biol Lett.

[33] Salviano-Silva A, Lobo-Alves SC, Almeida RC, Malheiros D, Petzl-Erler ML (2018). Besides pathology: Long non-coding RNA in cell and tissue homeostasis. Noncoding RNA.

[34] Toraih EA, Alghamdi SA, El-Wazir A, Hosny MM, Hussein MH, Khashana MS (2018). Dual biomarkers long non-coding RNA GAS5 and microRNA-34a co-expression signature in common solid tumors. PLoS One.

[35] Lu S, Su Z, Fu W, Cui Z, Jiang X, Tai S (2019). Altered expression of long non-coding RNA GAS5 in digestive tumors. Biosci Rep.

[36] Xie J, Herbert TP (2012). The role of mammalian target of rapamycin (mTOR) in the regulation of pancreatic beta-cell mass: Implications in the development of type-2 diabetes. Cell Mol Life Sci.

